# Leucine-rich repeat and sterile alpha motif containing 1 promotes the oncogenic growth of human hepatocellular carcinoma cells

**DOI:** 10.1186/s12935-019-0976-x

**Published:** 2019-10-03

**Authors:** Lili Pian, Xiaofeng Huang, Min Zhao, Yaolin Zhang, Cheng Qin, Jiyan Zhang, Jun Zhang, Qingyang Wang

**Affiliations:** 1 Beijing Institute of Brain Sciences, East 0749, 27# Taiping Road, Haidian District, Beijing, 100850 China; 2 Henan University Joint National Laboratory for Antibody Drug Engineering, School of Medicine, Henan University, Jinming District, Kaifeng, 475004 People’s Republic of China

**Keywords:** LRSAM1, Hepatocellular carcinoma, Tumorigenicity, Colony formation

## Abstract

**Background:**

Hepatocellular carcinoma (HCC), the most common primary cancer of the liver, is one of the most common malignancies and the leading cause of cancer-related death worldwide. Leucine-rich repeat and sterile alpha motif containing 1 (LRSAM1) is an E3 ubiquitin ligase involved in diverse cellular activities, including the regulation of cargo sorting, cell adhesion and antibacterial autophagy. The role of LRSAM1 in HCC remains unknown.

**Methods:**

In this study, we reviewed the TCGA database and then performed gain-of-function and loss-of-function analyses of LRSAM1 in HCC cell lines.

**Results:**

We found that the mRNA expression level of LRSAM1 was significantly increased in clinical HCC tissues in the TCGA database. Transient LRSAM1 knockdown in several human HCC cell lines led to reduced growth in conventional culture conditions. Stable LRSAM1 knockdown in HepG2 cells led to impaired anchorage-independent growth whereas its stable ectopic overexpression yielded the opposite effects. LRSAM1 overexpression in HepG2 cells enhanced in vivo tumorigenicity, whereas LRSAM1 knockdown in this cell line significantly impaired tumor growth.

**Conclusions:**

Our data suggest that LRSAM1 promotes the oncogenic growth of human HCC cells, although the underlying mechanisms remain to be explored.

## Background

Liver cancer remains a worldwide life-threatening disease that is the fifth most common in men and the ninth most common in women [1–3]. Hepatocellular carcinoma (HCC), accounting for approximately 70–85% of all liver cancers [4], is one of the most malignant tumors and the leading cause of cancer-related death worldwide [5–7]. Early stage-diagnosed patients have a relatively good prognosis with a 5-year survival rate over 70%; however, the majority of patients are diagnosed in the late-stage of the disease, and their overall 5-year survival rate is less than 16% [8]. The increasing incidence rate of HCC poses a serious challenge to many countries and regions [3].

Leucine-rich repeat and sterile alpha motif containing 1 (LRSAM1) is an E3 ubiquitin ligase involved in diverse cellular activities, including the regulation of cargo sorting, cell adhesion and antibacterial autophagy, which all depend on its E3 ligase function [9–11]. Ubiquitination is a pan existed process in cells specially for proteolysis of cellular proteins, which plays a pivotal role in maintenance of the balance between normal growth and uncontrolled proliferation [12]. This process is precisely regulated by a series of sequentially enzyme-mediated reactions that comprise of activation of ubiquitin by the activating enzyme (E1), holding ubiquitin by the conjugating enzyme (E2), and transfer of ubiquitin to target protein by the E3 ligase [13]. The cullin family of ubiquitin ligases represents the largest class of RING-type E3 ligases [14]. Recent report indicated that LRSAM1 plays a role in cellular bacteria clearance by recognizing bacteria via its LRR domain, followed by initiating autophagy in a RING domain-dependent manner [15].

Autophagy is a natural and regulated mechanism within the cell that disassembles unnecessary or dysfunctional components, allowing the orderly degradation and recycling of cellular components to maintain the cell itself [16]. Autophagic processes can be constitutive under normal conditions and can be an adaptive response under pathologic conditions, including stress, starvation, pathogen invasion and cancer [16, 17]. There is evidence indicating that autophagy plays an important role in tumor development, as both a tumor suppressor and a factor in tumor cell survival [18, 19]. Basic autophagy suppresses the malignant transformation of normal cells by maintaining genomic stability, whereas activated autophagy enables cancer cells to survive and develop under harsh circumstances [20, 21]. Additionally, the pro-survival or pro-death effects of autophagy on tumor cells also highly depend on the cancer type and the circumstance [22]. The role of autophagy in HCC progression is controversial because autophagy has been reported to promote the survival and proliferation of HCC cells [23, 24]; however, the opposite effects have also been observed [25].

Although it has been reported that the level of LRSAM1 is significantly increased in patients with colorectal cancer and can be downregulated by pectolinarigenin (PEC), a natural flavonoid from citrus fruits with antitumor effects in human gastric cancer cells [26, 27], the role of LRSAM1 in HCC remains unknown. In this study, we demonstrate for the first time that LRSAM1 promotes the oncogenic growth of human HCC cells. If autophagy is involved in LRSAM1 function, further investigation is needed.

## Methods

### Plasmids, small-interfering RNAs (siRNAs), and short hairpin RNAs (shRNAs)

A mammalian expression vector encoding GFP-LRSAM1 was generated by cloning PCR-amplified products of the *Lrsam1* gene into the pEGFP-N1 vector and confirmed by DNA sequencing. Human LRSAM1 siRNAs (177# GCTGATCGTCCACACGAAT, 712# CCCACGGACAGATTCTCAA) and non-targeting control (NC) siRNA were from Shanghai GenePharma (Shanghai, China). Lentivirus-based human LRSAM1 shRNAs (549# GCTGATCGTCCACGAATCA, 1636# GCCGAAATGGATGAACGATTC) and NC shRNA were from Shanghai GenePharma (Shanghai, China).

### Antibodies and reagents

Antibodies against LRSAM1 (#24666-1-AP) and LC3B (#14600-1-AP) were purchased from Proteintech. Antibodies against β-actin (#ab8334) were purchased from Abcam. Horseradish peroxidase-conjugated goat anti-rabbit (#ZB-2301) and goat anti-mouse IgG (#ZB-2305) were purchased from Beijing Zhongshan Jinqiao Biotechnology Co., Ltd. Lipofectamine 2000, Lipofectamine RNAiMAX, neomycin, and hygromycin were purchased from Invitrogen. Agar, MTT dry powder, RNase A, and PI were purchased from Sigma.

### Western blot analysis

Cells were lysed and homogenized in RIPA buffer (50 mM Tris–HCl, pH 7.5, 1% NP40, 0.35% DOC, 150 mM NaCl, 1 mM EDTA, and 1 mM EGTA) supplemented with protease and phosphatase inhibitor cocktails. Whole cell lysates were subjected to SDS-PAGE separation on 12% acrylamide gels, followed by transfer onto PVDF membranes for 3 h. After blocking with 5% nonfat milk-containing TBS-Tween-20 buffer, the blots were incubated with primary antibodies overnight at 4 °C, followed by incubation with HRP-conjugated secondary antibodies for 1 h at room temperature. The immunoreactive bands were visualized with an enhanced chemiluminescence (ECL) detection reagent.

### Cell culture and transfection

Human HCC cell lines (HepG2, BEL-7404, Huh7, and SK-hep1) were purchased from the Shanghai Institutes for Biological Sciences. All cell lines were cultured in Dulbecco’s modified Eagle’s medium supplemented with 10% fetal bovine serum, 100 U/mL penicillin, and 100 μg/mL streptomycin and were maintained at 37 °C with 5% CO_2_. Transfection was performed with Lipofectamine 2000 or Lipofectamine RNAiMAX. Stable clones were selected with 600 μg/mL neomycin (Invitrogen) or 1 μg/mL hygromycin for approximately 2 months.

### Cell cycle analysis

A total of 1 × 10^6^ cells were harvested and fixed in 75% cold ethanol for at least 18 h. Then, the cells were digested with RNase A (10 μg/mL, 30 min) at 37 °C, labeled with PI (50 μg/mL, 30 min) at room temperature in the dark, and analyzed by flow cytometry. Flow cytometry was carried out on a Becton–Dickinson FACSCalibur (BD Biosciences).

### Soft agar colony formation assay

Soft agar colony formation assays were performed with agar gels in 6-well plates. The bottom layer (1.5 mL per well) was prepared by mixing 2× DMEM, 1.2% agar and serum at a ratio of 4.5 mL:4.5 mL:1 mL. Then, the middle layer (1.5 mL per well) was prepared by mixing 2× DMEM, serum, single-cell suspensions and 0.6% gel at a ratio of 1400 μL:350 μL:350 μL:1400 μL. Once the middle layer had solidified, 1 mL 1× DMEM was gently added into the 6-well plates. After 3 weeks, MTT was added to the medium to visualize the colonies.

### In vivo tumor growth

Male athymic BALB/c nude mice were purchased from Beijing Vital River Laboratory Animal Technology Co., Ltd. and maintained under specific pathogen-free conditions. All experiments were performed in accordance with institutional guidelines for animal care. 6- to 8-week-old nude mice were inoculated subcutaneously with HepG2 cells (1 × 10^6^/0.2 mL phosphate-buffered saline, n = 6–7). The length and width of the tumors were measured with calipers at the indicated time points.

### Statistical analyses

All experiments were carried out at least three times. The data are presented as the mean ± standard deviation. Statistical analysis was conducted using the SPSS 13.0 statistical software package. For comparisons between two groups, Student’s *t*-test was used. *P* < 0.05 was considered to be statistically significant.

## Results

### Up-regulation of LRSAM1 mRNA in human HCC

**Fig. 1:**
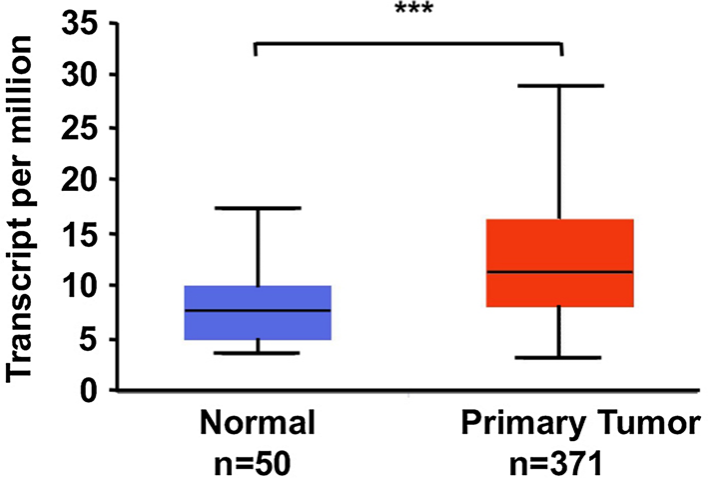
Up-regulation of LRSAM1 mRNA in human HCC according to the TCGA database. *P < 0.05; **P < 0.01; ***P < 0.001; ns, no significance

UALCAN is a website-based TCGA database that allows the analysis of gene expression via tumor histology. By referring to the TCGA database, we found that the expression levels of LRSAM1 mRNA are significantly higher in HCC liver tissues than in normal liver tissues. This difference is not affected by factors such as age, sex, race, weight, cancer stage or tumor grade (Fig. 1 and data not shown).

### LRSAM1 promotes the growth of human HCC cells in conventional culture conditions

**Fig. 2:**
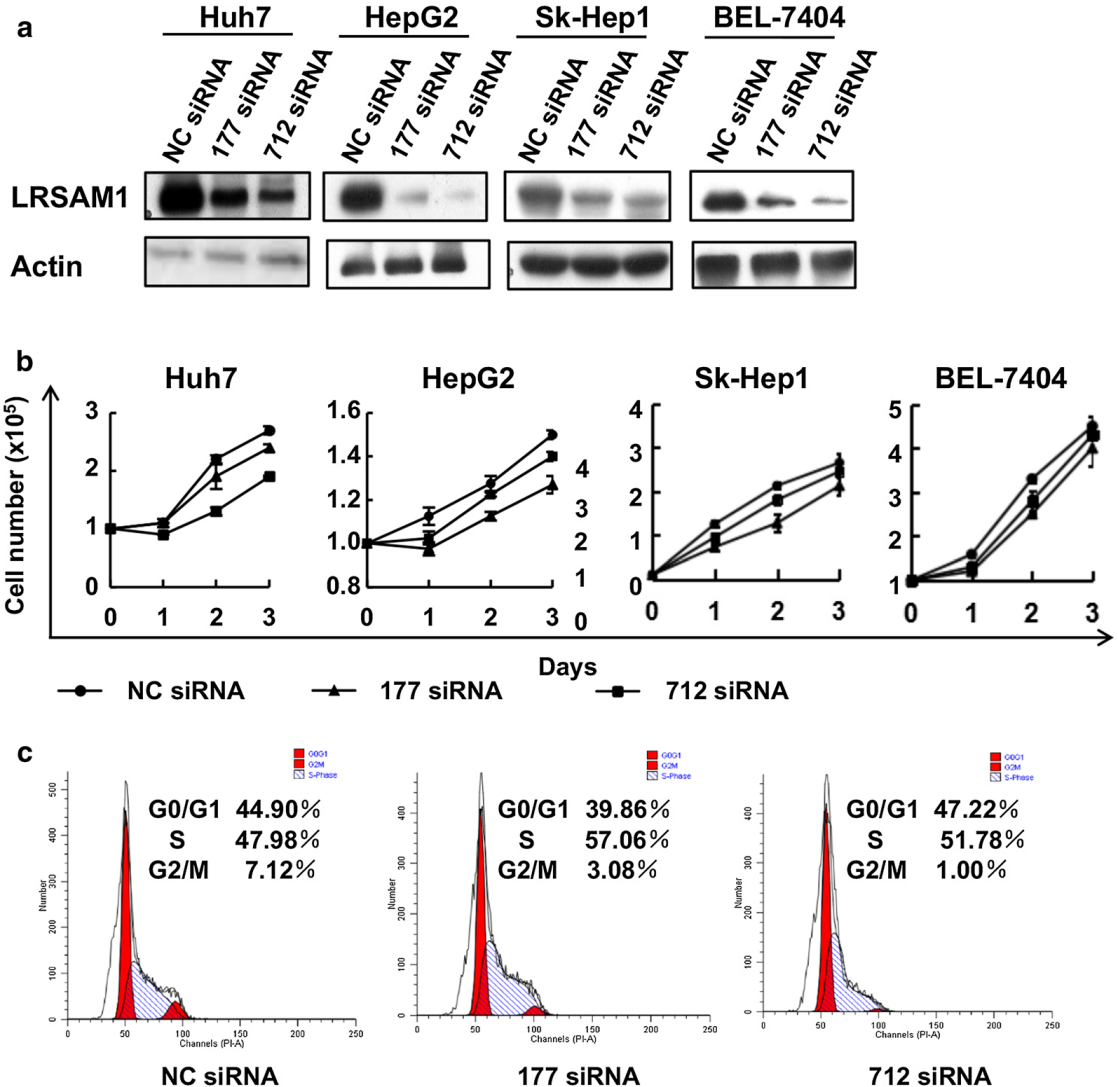
LRSAM1 promotes the growth of human HCC cells in conventional culture conditions. Human HCC cell lines (Huh7, HepG2, Sk-Hep1 and BEL-7404) were transfected with non-targeting control (NC) siRNA or LRSAM1 siRNAs. Forty-eight hours later, the cells were subjected to Western blot analysis for LRSAM1 expression (**a**), cell counting daily for 3 consecutive days to examine the growth rate (**b**), and cell cycle analysis (**c**). The data are shown as the mean ± SD, n = 3

In this study, we downregulated LRSAM1 expression using siRNAs. First, Western blot analysis revealed that LRSAM1 siRNAs could efficiently knock down endogenous LRSAM1 in all 4 HCC cell lines (Huh7, BEL-7404, SK-Hep1, and HepG2) (Fig. 2a). Based on siRNA-mediated knockdown, cell counting revealed that LRSAM1 knockdown led to reduced growth in conventional culture conditions (Fig. 2b). Cell cycle analysis revealed a decreased percentage of G2/M phase cells but no increase in sub-G1 phase cells (Fig. 2c). These data suggest that LRSAM1 enhances the growth of human HCC cells in conventional culture conditions by promoting cell cycle progression but not cellular survival.

### LRSAM1 promotes the anchorage-independent growth of human HCC cells

**Fig. 3:**
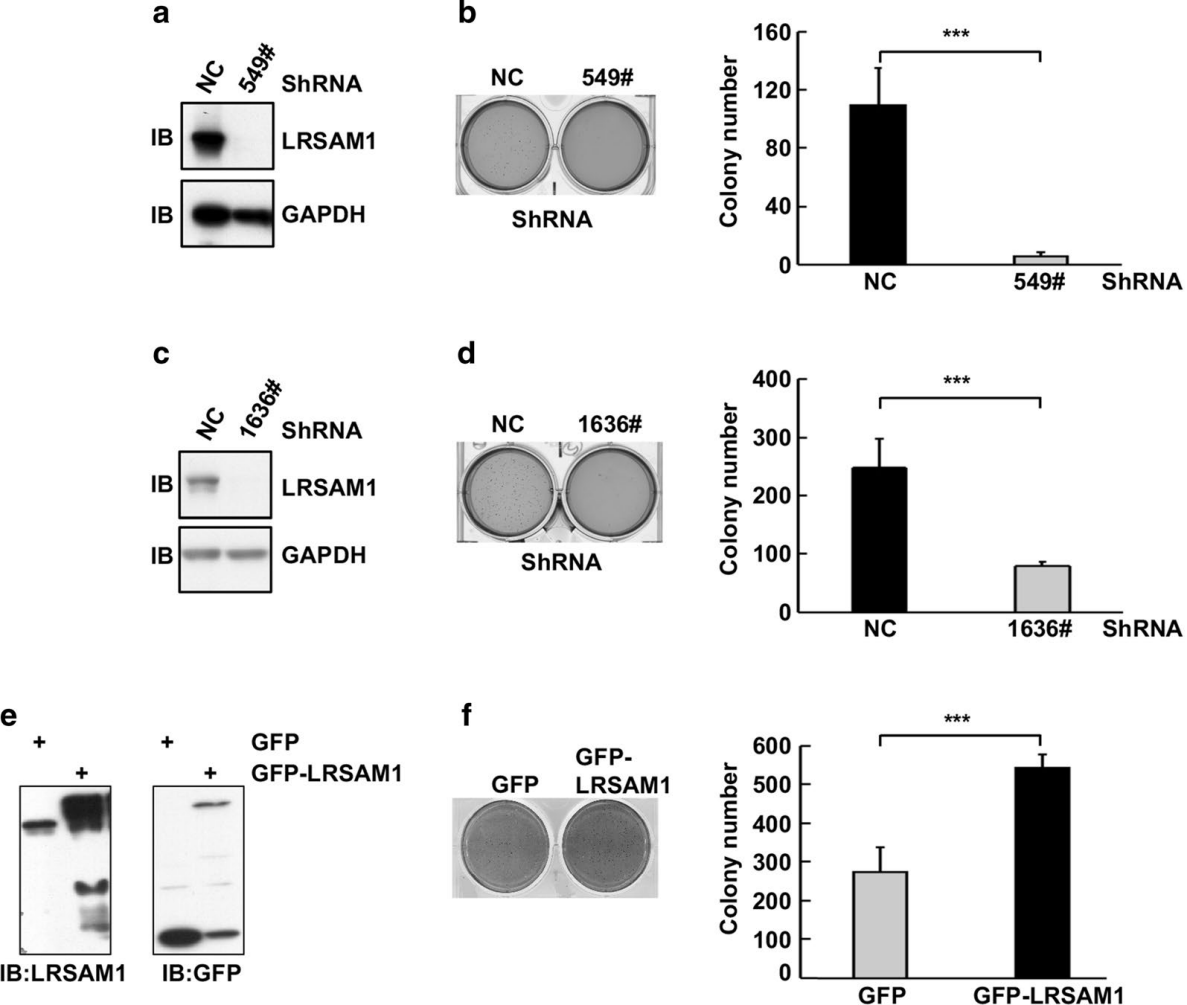
LRSAM1 promotes the anchorage-independent growth of human HCC cells. HepG2 single clones stably expressing control shRNA or LRSAM1 shRNAs (**a**–**d**) or HepG2 mixed clones stably expressing GFP or GFP-LRSAM1 were generated. The cells were subjected to Western blot analysis for LRSAM1 expression (**a**, **c**, **e**) and soft agar assays to examine the colony forming ability (**b**, **d**, **f**). The data are shown as the mean ± SD, n = 3; *P < 0.05; **P < 0.01; ***P < 0.001; ns, no significance

Malignant cells can survive and form colonies in soft agar, which mimics the oncogenic growth of tumor cells better than conventional culture conditions. To explore how LRSAM1 might affect the colony forming ability of human HCC cells, we generated HepG2 single clones stably expressing control shRNA or LRSAM1 shRNA 549#. Western blot analysis confirmed the efficient knockdown (Fig. 3a). Soft agar assays showed that stable silencing of endogenous LRSAM1 resulted in fewer colony numbers (Fig. 3b). To confirm this, we generated another set of HepG2 single clones stably expressing control shRNA or LRSAM1 shRNA 1636#. Consistently, fewer colonies were observed upon LRSAM1 stable knockdown (Fig. 3c, d). Next, we generated HepG2 mixed clones stably expressing GFP or GFP-LRSAM1 (Fig. 3e). As expected, LRSAM1 overexpression enhanced the colony forming ability (Fig. 3f). Thus, LRSAM1 promotes the anchorage-independent growth of human HCC cells.

### LRSAM1 promotes the in vivo tumorigenicity of human HCC cells

**Fig. 4:**
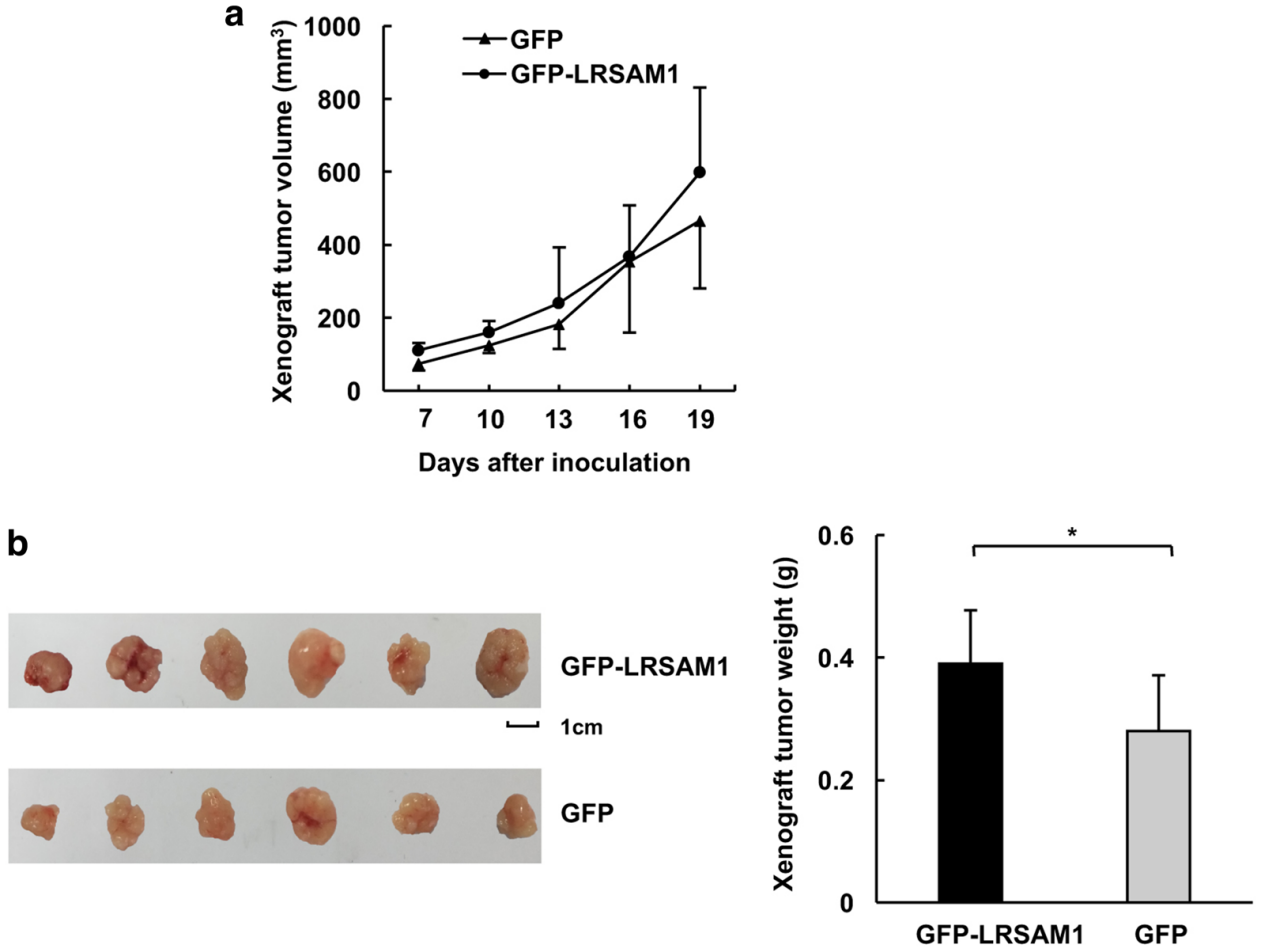
LRSAM1 overexpression promotes the in vitro tumorigenicity of human HCC cells. HepG2 mixed clones stably expressing GFP or GFP-LRSAM1 were injected into nude mice. After various times as indicated, the tumors were measured with Vernier calipers (mean ± SD) (**a**). Images (*left panel*) and weights (*right panel*) of the subcutaneous tumors after the mice were sacrificed are shown (**b**). The data are shown as the mean ± SD, n = 3; *P < 0.05; **P < 0.01; ***P < 0.001; ns, no significance

**Fig. 5:**
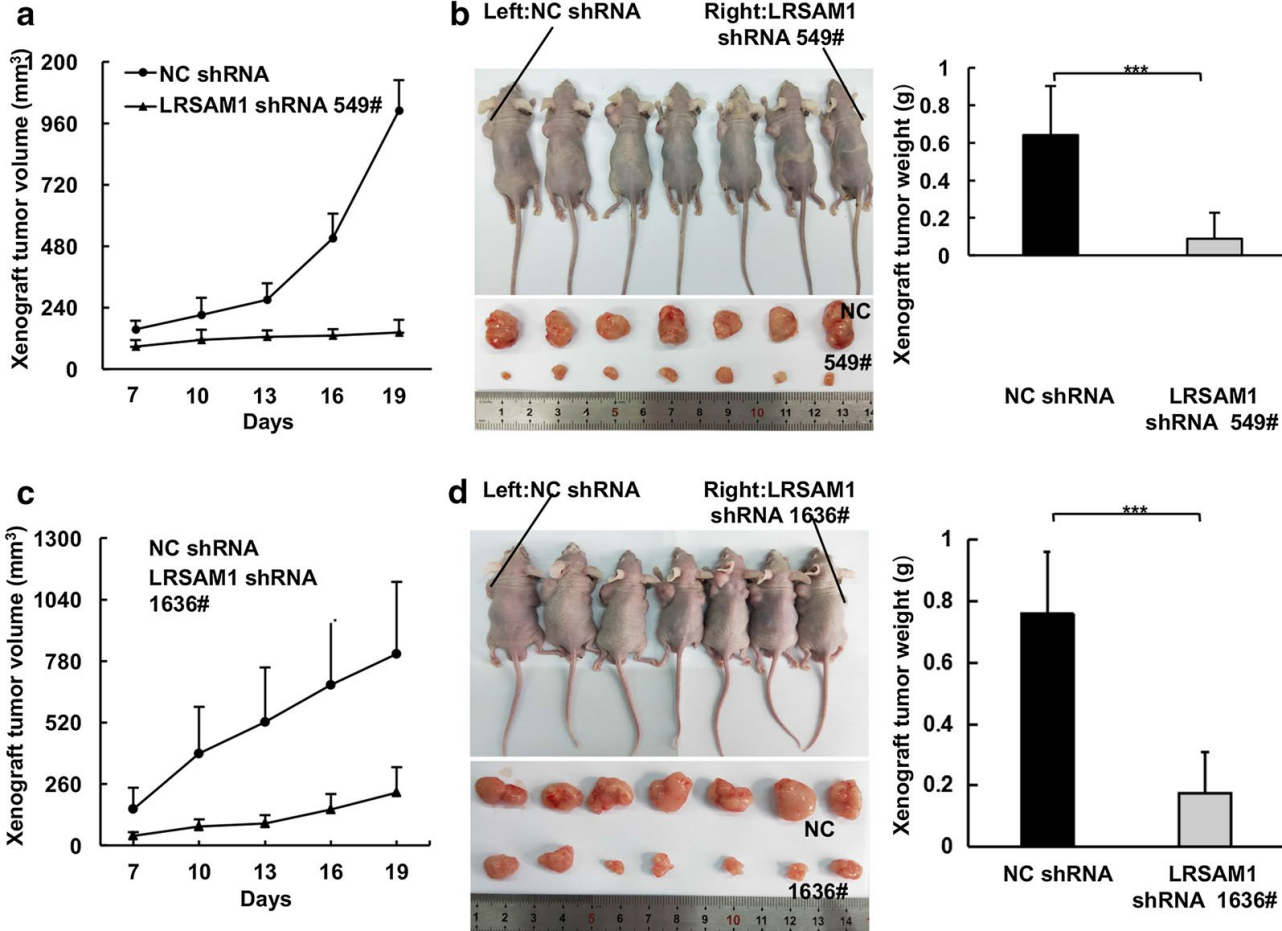
LRSAM1 knockdown impairs the in vitro tumorigenicity of human HCC cells. HepG2 single clones stably expressing control shRNA or LRSAM1 shRNAs were injected into nude mice. After various times as indicated, the tumors were measured with Vernier calipers (mean ± SD) (**a**, **c**). Images (*left panel*) and weights (*right panel*) of the subcutaneous tumors after the mice were sacrificed are shown (**b**, **d**). The data are shown as the mean ± SD, n = 3; *P < 0.05; **P < 0.01; ***P < 0.001; ns, no significance

Then, we employed subcutaneous tumor formation in nude mice to explore how LRSAM1 might affect the tumorigenic growth of human HCC cells in vivo. The results demonstrated that LRSAM1 overexpression moderately enhanced tumor growth, as revealed by the tumor growth curves (Fig. 4a) and xenograft weights (Fig. 4b). More importantly, stable silencing of endogenous LRSAM1 by shRNAs targeting different sequences resulted in dramatic decrease in tumor growth, which was indicated by the tumor growth curves (Fig. 5a, c) and xenograft weights (Fig. 5b, d).

## Discussion

HCC is a common malignant tumor that is now the third-highest cause of tumor death, seriously threatening human life and health [28]. Previous studies have shown that LRSAM1 expression levels are significantly increased in colorectal cancer patients, suggesting that abnormal LRSAM1 expression may be involved in cancer progression [26]. By querying TCGA databases, we found that the expression of LRSAM1 in HCC patients was significantly increased, indicating that LRSAM1 plays an important role in liver tumorigenesis. By exploring the effects of LRSAM1 on liver cancer cell tumorigenicity, our data may provide a new therapeutic option for HCC patients.

Our study suggests that LRSAM1 promotes the oncogenic growth of human liver cancer cells. Interestingly, LRSAM1 overexpression in HepG2 cells only moderately enhanced in vivo tumorigenicity, whereas LRSAM1 knockdown in this cell line significantly impaired tumor growth. This may be because HepG2 cells express high level of endogenous LRSAM1.

Furthermore, our preliminary data suggest that LRSAM1 enhances the growth of human HCC cells by promoting cell cycle progression but not cellular survival. However, how does LRSAM1 promote the proliferation of human HCC cells? Previous studies have indicated that LRSAM1 has been considered an important regulator of autophagy against bacteria via its LRR and RING domains [29], and LRSAM1 promotes autophagy and enhances autophagosomes formation [15, 16]. In this study, the Western blot data revealed that LC3BII, an indicator of autophagy, was significantly increased upon LRSAM1 knockdown, while overexpression of LRSAM1 led to the opposite effects (Additional file 1: Figure S1). The role of autophagy in cancer progression is complicated [30, 31]. Furthermore, the role of autophagy in LRSAM1-mediated malignant proliferation in human HCC cells remains elusive, and a large number of experimental studies are needed to reveal this in the future.

## Conclusions

Our data first suggest that LRSAM1 promotes the oncogenic growth of human HCC cells, and knock down of LRSAM1 suppresses the malignant growth of HCC xenograft in nude mice. We propose LRSAM1 as a therapeutic target for the clinical treatment of HCC.

## Supplementary information

**Additional file 1: Figure S1.** LRSAM1 knockdown promotes autophagy in human HCC cells. HepG2 cells transiently expressing control siRNA or LRSAM1 siRNAs (left), stably expressing control shRNA or LRSAM1 shRNA (middle), or transiently expressing GFP or GFP-LRSAM1 (right) were harvested and subjected to Western blot analysis of LC3B and GAPDH.

## Data Availability

UALCAN is a website-based TCGA database that allows the analysis of gene expression via tumor histology. Check the link below: http://ualcan.path.uab.edu/analysis.html.

## Abbreviations

HCC: hepatocellular carcinoma
LRSAM1: leucine-rich repeat and sterile alpha motif containing 1
LRR: leucine-rich repeat
PEC: pectolinarigenin
siRNAs: small-interfering RNAs
shRNAs: short hairpin RNAs
