# Supplementary figures and images for: Leucine-rich repeat and sterile alpha motif containing 1 promotes the oncogenic growth of human hepatocellular carcinoma cells
Source: Cancer Cell Int. 2019 Oct 3;19:255. doi: 10.1186/s12935-019-0976-x (PMC6775663; doi:10.1186/s12935-019-0976-x)

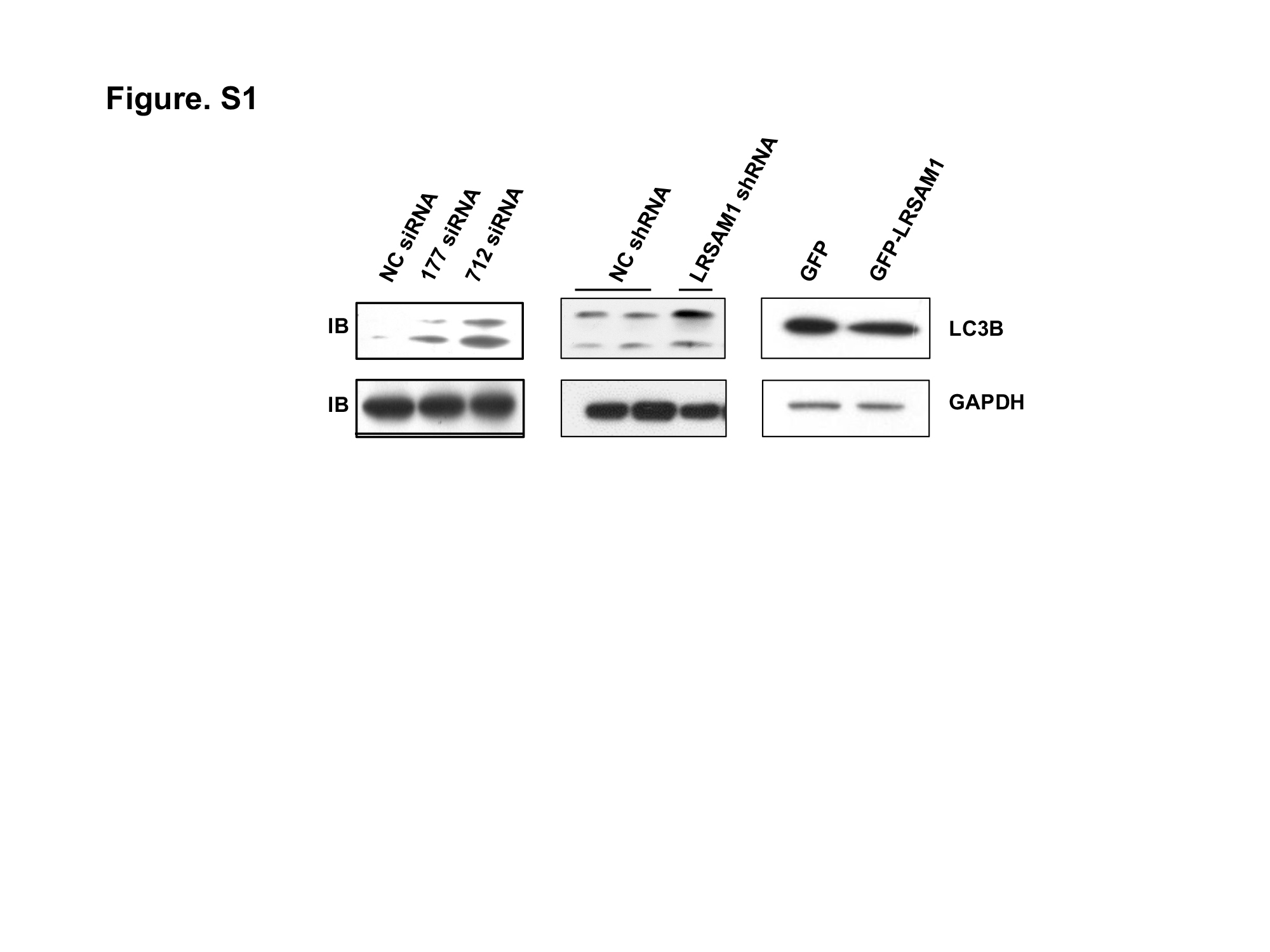

Supplement: Supplementary file 1 — Additional file 1: Figure S1. LRSAM1 knockdown promotes autophagy in human HCC cells. HepG2 cells transiently expressing control siRNA or LRSAM1 siRNAs (left), stably expressing control shRNA or LRSAM1 shRNA (middle), or transiently expressing GFP or GFP-LRSAM1 (right) were harvested and subjected to Western blot analysis of LC3B and GAPDH. [file 12935_2019_976_MOESM1_ESM.jpg]
